# Assessment of soil property in the Guyuan region from Ningxia Province of China and prediction of pepper blight

**DOI:** 10.1371/journal.pone.0293173

**Published:** 2023-11-20

**Authors:** Yi Hou, Yu-Long Ma, Xiao-Min Wang, Guo-Xin Cheng

**Affiliations:** School of Wine and Horticulture, Ningxia University, Yinchuan, Ningxia, P. R. China; Nuclear Science and Technology Research Institute, ISLAMIC REPUBLIC OF IRAN

## Abstract

Soil quality is an important determinant of soil-use efficiency in the Loess Plateau. However, there is no in-depth study on the soil quality of the Loess Plateau. The present study compared the quality of the 0–20 cm soil layer (T0-20) and the 20–40 cm soil layer (T20-40) from the Guyuan region located in the Loess Plateau. The analysis revealed that T0-20 had a higher content of total N, total P, available P, and organic matter, and the activities of microbial enzymes, especially β-grape-glycosidase (β-GC) and sucrase (SC), than T20-40, indicating that soil quality in T0-20 was better than T20-40. Amplicon sequencing found that Pseudombrophila from Ascomycota was the most abundant microbial species and significantly differed between T0-20 (34.2%) and T20-40 (48.7%). This species and another 19 microbial species, such as Ceratobasidiaceae and Mortierellaceae, determined the diversity of soil microorganism. Further analysis of the phenotype and other parameters of pepper seedlings subjected to *P*. *capsici* infection isolated from test soil revealed that decreased organic matter content in deep soil layer is related to happening of pepper blight, and 3 h after infection was the critical time point for infection. The peroxidase (POD) activity increased after *P*. *capsici* infection and was positively correlated with infection time, suggesting this enzyme may be an indicator of pepper blight occurrence. These findings provide a theoretical foundation for planning pepper blight management and crop cultivation strategies in the Guyuan region.

## 1 Introduction

In 1972, the United Nations Food Development Agency identified the Guyuan region in China as one of the most inhospitable regions for human survival [1]. This region is located in the Loess Plateau of northwest China, which lacks rain and where the sand storm wreaked havoc [2, 3]. Loess is the main soil structure in this region and is characterized by low water retention capacity and serious salinization [4, 5]. Therefore, crops in this region demonstrate poor growth and low yield, leading to a failure to satisfy people’s survival needs. Thus, an improvement in the soil-use efficiency of this region is important.

Soil quality can be reflected by organic content, microbial diversity, soil enzyme activity, macroelement content and pH, and plays a key role in maintaining the plant-soil-microbial balance [6, 7]. Activity and levels of microbial enzymes, including urease (UE), polyphenol oxidase (PPO), α-glucosidase (α-GC), β-glucosidase (β-GC), cellulase (CL), catalase (CAT), sucrase (SC), peroxidase (POD), alkaline phosphatase (AKP/ALP), and alkaline phosphatase (ALPT), can improve soil permeability, chemical composition, and organic matter content [8–10]. This indicates that there is a positive correlation between soil microbial biomass and soil quality [11]. These microbial enzymes, found in the solid or liquid phase of soil, participated in the decomposition and synthesis of soil organic matter and the circulation of N, phosphorus (P), potassium (K), and other substances [12, 13]. Therefore, research on soil biological activity and fertility is important to understand soil quality in the field.

Pepper (*Capsicum annuum* L.) is an agriculturally important vegetable in the Solanaceae family [14, 15]. Owing to its unique cold climate advantages, Ningxia has become an important pepper-producing area in Northwest China. As a result, pepper cultivation in Ningxia Province has steadily increased and created considerable economic value since 2000 (based on the government report data) However, during the growing season, pepper is easily and frequently infected by *Phytophthora capsici* (*P*. *capsici*), severely reducing the yield and economic value [16]. *P*. *capsici* is a typical soil-borne pathogen infecting various tissues/organs of pepper plants [17]. Under adverse conditions, *P*. *capsici* results in secondary diseases in pepper at the later stage of growth, enhancing crop management cost and reducing the economic benefits [18]. During evolution, pepper plants have evolved certain defense mechanisms, such as the accumulation of antioxidant enzymes [19]. Typically, pathogen infection causes an imbalance in reactive oxygen species (ROS) and triggers the accumulation of peroxidase (POD), superoxide dismutase (SOD), catalase (CAT), and ascorbate oxidase (APX) [20, 21]. These antioxidant enzymes neutralize the harmful effects of ROS and can be quantified by colorimetric signals [22, 23]. Therefore, the antioxidant enzymes indicate the occurrence of plant diseases.

The Loess Plateau of northwest China has been considered an underdeveloped region with a large population due to the difficulty in producing the daily necessities using the limited and poor soil resources is a major concern in this region. Several researchers have assessed soil fertility and microbial diversity of various regions; however, soil chemistry, soil biology, and related disciplines have not been analyzed to form a consistent, quantitative evaluation index to characterize soil quality including soil chemical property and microorganism diversity. Therefore, the present study analyzed the nutrition, soil enzyme activity, and microbial diversity in the soils of the Guyuan region and assessed the critical time and indicator of pepper blight. Our study will provide an effective management strategy for pepper cultivation in this region.

## 2 Materials and methods

### 2.1 Experimental materials

Soil samples used in this study were obtained from the 0–20 cm (T0-20) and 20–40 cm (T20-40) depths from the Guyuan region of Ningxia Province in China (N 105°72’, S 35°96’). Ningxia is located in the semi-arid region of China with less than 500 mm of rain. Sandy-textured soil is the main soil type in this region, and its characteristics include the lack of effective water retention capacity and severe salinization. Nutrient loss and plant diseases are the major issues limiting the sustainable utilization of the soil in this area as a result of crop rotation and fertilizer application for a number of years. Each sample was an equal mixture of soil from 7 sampling locations in this area (S1 Fig). The pepper cultivars ‘EC’ (susceptible to PC-1 strain of *P*. *capsici*) and ‘AA5’ (resistant to PC-1 strain of *P*. *capsici*) were used in this study. ‘PC-1’ was a virulent strain of *P*. *capsici* isolated from pepperplants. The strong pathogenic strain of *P*. *capsici* ‘PC-1’ was provided by the Horticulture Group of Ningxia University. In addition, weather parameters was measured at 19 days before the soil was sampled using the automatic meteorological station (V BK-QC4, Bokey, Shandong).

### 2.2 Enzymes activities and nutrients in the soil

Approximately 0.25 g of dry soil was sampled to analyse the soil pH, available nitrogen (N), total N, available phosphorus (P), total P, and organic content following the reported methods [24]. Meanwhile, 2.0 g of fresh soil from T0-20 and T20-40 were used to measure the activities of soil enzymes, including urease (UE), polyphenol oxidase (PPO), α-glucosidase (α-GC), β-grape-glycosidase (β-GC), cellulase (CL), catalase (CAT), sucrase (SC), peroxidase (POD), alkaline phosphatase (AKP/ALP), and alcalase protease (ALPT), using the corresponding kits obtained from JICE Biotechnology Co., LTD. (Nanjing, China). Three independent experiments were carried out to analyze the soil fertility and microbial enzyme activity. All kits are listed in S1 Table.

### 2.3 Amplicon sequencing-based detection

To analyze microbial composition, the amplicon sequencing was performed in this study. Approximately 0.5 g of the soil sample was used to extract DNA following the cetyl trimethyl ammonium bromide (CTAB) method [25]. The extracted DNA was further purified using the DNA purification kit (Yeasen Biotechnology Co., Ltd., Shanghai, China). Then, 16S rRNA, 18S rRNA, and ITS genes were amplified using specific primers (16S V4: 515F/806R, 18S V4: 528F/706R, 18S ITS1/ITS2) (S2 Table). All PCR reactions were carried out using the PCR Master Mix (Phusion^®^ High -Fidelity, New England Biolabs Company, England), following the manufacturer’s instructions and using the following program: initial denaturation at 98°C for 1 min, followed by 30 cycles of denaturation at 98°C for 10 s, annealing at 50°C for 30 s, and elongation at 72°C for 30 s, and final extension at 72°C for 5 min. PCR products from each sample were mixed in equidensity ratios, and the pooled product was purified with Qiagen Gel Extraction Kit (Qiagen, Germany). Sequencing libraries were generated using TruSeq ^®^ DNA PCR-Free Sample Preparation Kit (Illumina, USA), following the manufacturer’s recommendations, and subsequently, the index codes were added. The library quality was assessed using the Qubit^@^ 2.0 Fluorometer (Thermo Scientific) and Agilent Bioanalyzer 2100 system (Agilent, USA). Finally, the library was sequenced on an Illumina NovaSeq platform to generate the paired-end reads.

### 2.4 Data analysis

The paired-end reads were assigned to samples and merged using FLASH (V1.2.7, http://ccb.jhu.edu/software/FLASH/), which merges the reads when the correct overlap is found; the splicing sequences were called the raw tag. Quality filtering on the raw tags was performed using QIIME (V1.9.1, http://qiime.org/scripts/split_libraries_fastq.html) under specific filtering conditions to obtain high-quality clean tags. The tags were then compared with the reference database [Silva database (16S/18S), https://www.arb-silva.de/; Unite Database (ITS), https://unite.ut.ee/] using UCHIME Algorithm (http://www.drive5.com/usearch/manual/uchime_algo.html) to detect and remove the chimera sequences and finally obtain the effective tags.

Sequences analysis was performed using the Uparse software (Uparsev7.0. 1001, http://drive5.com/uparse/). Sequences with ≥97% similarity were assigned to the same Operational Taxonomic Units (OTUs). For each representative sequence of 16S, 18S, and ITS, the Silva Database (http://www.arb-silva.de/) was used based on Mothur algorithm, RDP classifier algorithm, and BLAST algorithm, respectively, for taxonomic annotation. The representative sequence for each OTU was screened for further annotation. Multiple sequence alignment was performed using the MUSCLE software (V3.8.31, http://www.drive5.com/muscle/). OTUs abundance information was normalized using a standard of sequence number corresponding to the sample with the least sequences. Alpha diversity and Beta diversity were measured using the QIIME software (V1.9.1) to assess and evaluate the differences between T0-20 and T20-40 in microbial species complexity.

### 2.5 Predicting *P*. *capsici* occurrence

The virulent ‘PC-1’ strain of *P*. *capsici* was isolated from the test soil and used in this experiment to infect the pepper plants The *P*. *capsici* zoospore suspension was prepared according to the protocol by Zhang et al. 2016 [26]. The virulent ‘PC-1’ strain was grown on potato dextrose agar (PDA) for seven days in the dark at 28°C. The leaf veins were used as the dividing line, agar plugs of the media with the strain were placed on the right side of the leaf veins, while another side do same treatment with water instead of agar plugs of the media as the control. These control and treated leaves were collected at 0 h, 1 h, 3 h, 6 h, 12 h, and 24 h after inoculation. Three independent experiments were conducted, and three pepper seedlings were maintained per treatment., the right side was inoculated with Phytophthora, the left side was used as the control.

### 2.6 Determination of antioxidant enzyme activity and MDA content

Approximately 0.2 g of the leaf was sampled and ground in a mortar with 5 mL of phosphate buffer to determine the content of malondialdehyde (MDA) and the activities of superoxide dismutase (SOD), peroxidase (POD), and catalase (CAT). These components were quantified spectrophotometrically at specific optical densities (ODs) as described by Dionisio-Sese & Tobita. 1998 [27].

### 2.7 Quantitative real-time PCR analysis (qPCR)

Total RNA was extracted using the Trizol reagent and was converted into first-strand cDNA using a PrimeScript Kit (Takara, Dalian, China). The resulting cDNA was used as a template for quantitative PCR amplification using SYBR as the fluorescent reporter. Fibe key genes encoding PODs, including *CaPOD2*(LOC10787408*2)*, *CaPOD10* (LOC107860325), *CaPOD41* (LOC107850917), *CaPOD42* (LOC107859584), *POD45* (LOC107856092) and *CaPOD51* (LOC107860206), were assessed using qRT-PCR. Using information from GenBank, primers were designed, and their sequences are listed in the S2 Table. qRT-PCR was conducted using the iQ5 system (BIO-RAD, USA). The 2^-△△CT^ method was used to analyze the relative transcript expression levels using the means from three replicates.

### 2.8 Statistical analysis

All contributed parameters were measured with three replicates, and the Data were evaluated by an analysis of variance (ANOVA) using SPSS (V16.0, SPSS Inc., Chicago, IL, USA). All parameter values are expressed as the means ± standard deviation (SD) of three replicates. A least significant difference (*P* ≤ 0.05) test was used to identify statistically significant differences among the treatment means.

## 3 Results

### 3.1 Nutrient content and microbial activities in soil

**Fig 1:**
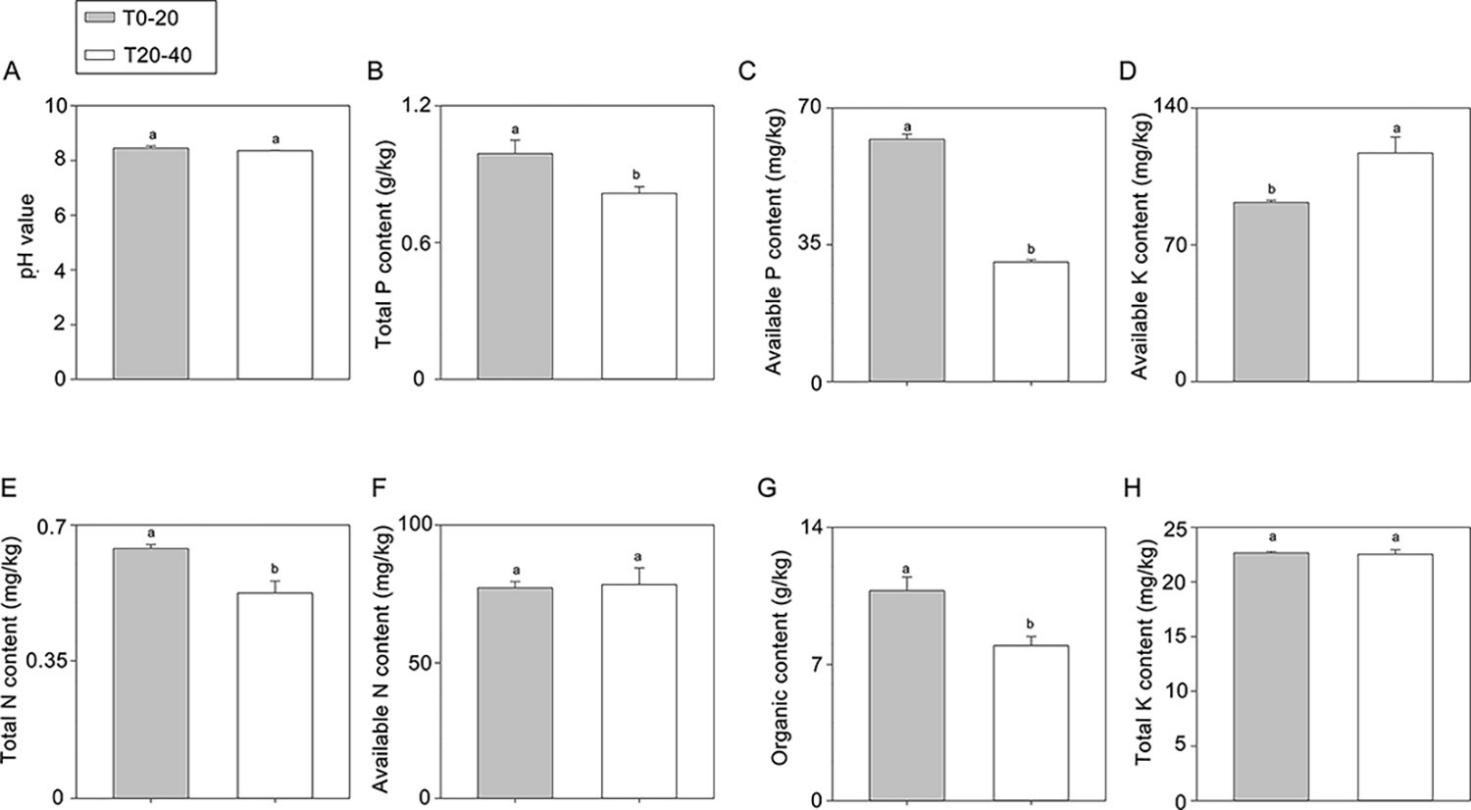
Nutrient content in different layers of soil. (A) pH value (B) total P content (C) available P content (D) available K content (E) total N content (F) available N content (G) organic content (H) total K content. 0–20 cm and 20–40 cm layers of soil were sampled for this experiment, respectively. Each soil sample contains three repeats. The error bars represent SD for three biological replicates, and the lowercases showed the significant level at *P* < 0.05.

Fig 1 shows the difference in pH value and nutrition components in T0-20 and T20-40. The pH values of the two soil samples were greater than 8.0, suggesting that the Loess in the Guyan region has typical alkaline soil. The analysis also revealed significant differences in total N, total P, available P, and organic content between T0-20 and T20-40 (Fig 1B, 1C, 1E and 1G); however, no significant difference was observed in the total K and available N content (Fig 1F and 1H). This observation indicated that the soil effective component in T0-20 is better than in T20-40.

**Fig 2:**
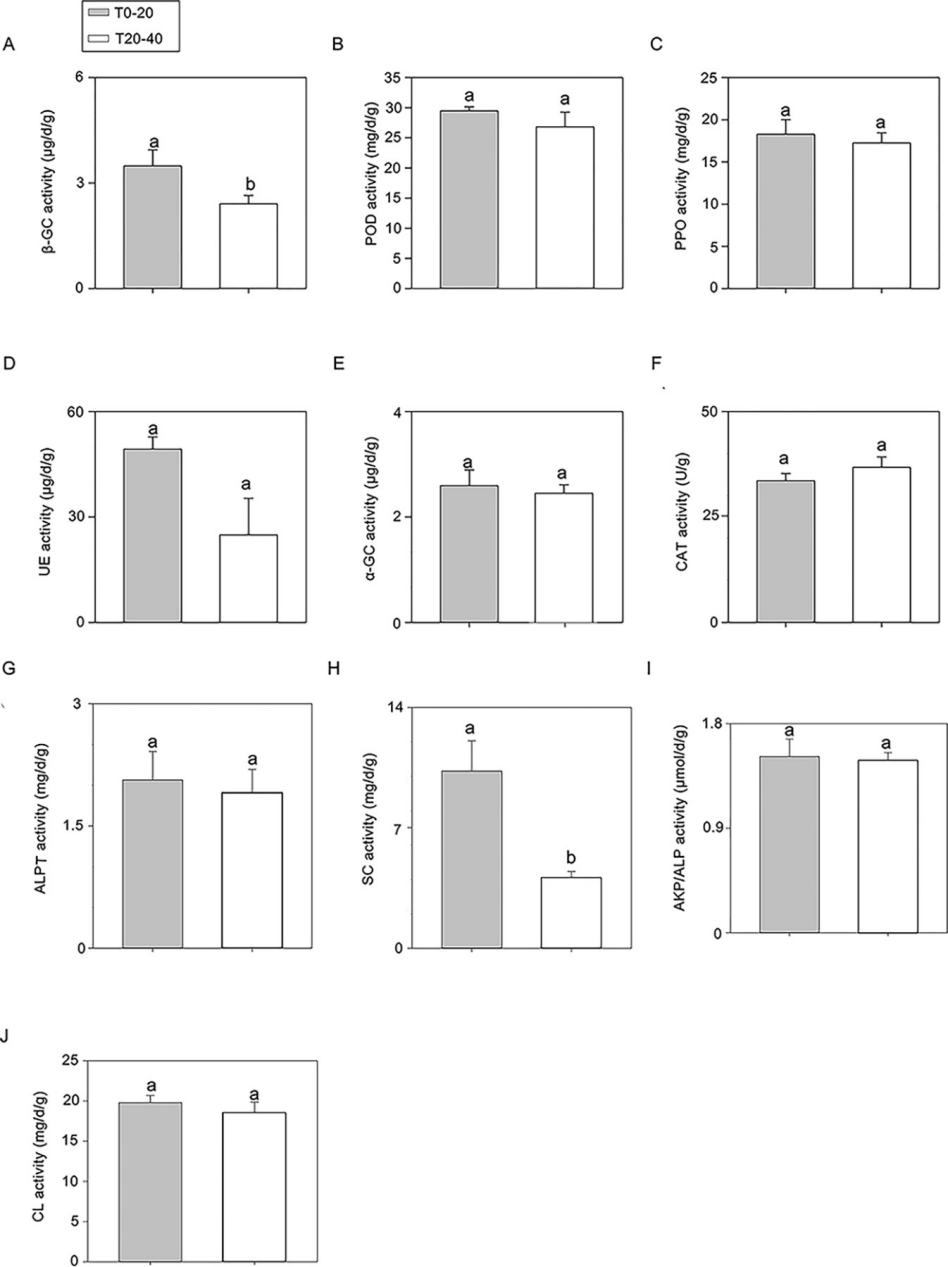
The levels of microbial enzyme activities in T0-20 and T20-40. (A) β-GC activity (B) POD activity (C) PPO activity (D) UE activity (E) α-GC activity (F) CAT activity (G) ALPC activity (H) SC activity (I) AKP/ALP activity (J) CL activity. 0–20 cm and 20–40 cm layers of soil were sampled for this experiment, respectively. Each soil sample contains three repeats. The error bars represent SD for three biological replicates, and the lowercases showed the significant level at *P* < 0.05.

With the increase in soil depth, the activity of microbial enzymes gradually decreased. The activities of all enzymes in T0-20 were higher than that in T20-40; significant differences were detected in β-GC and SC activities between T0-20 and T20-40. These results suggest that T0-20 possessed higher soil enzyme activities than T20-40 (Fig 2).

### 3.2 Soil microbial diversity and functional network

**Fig 3:**
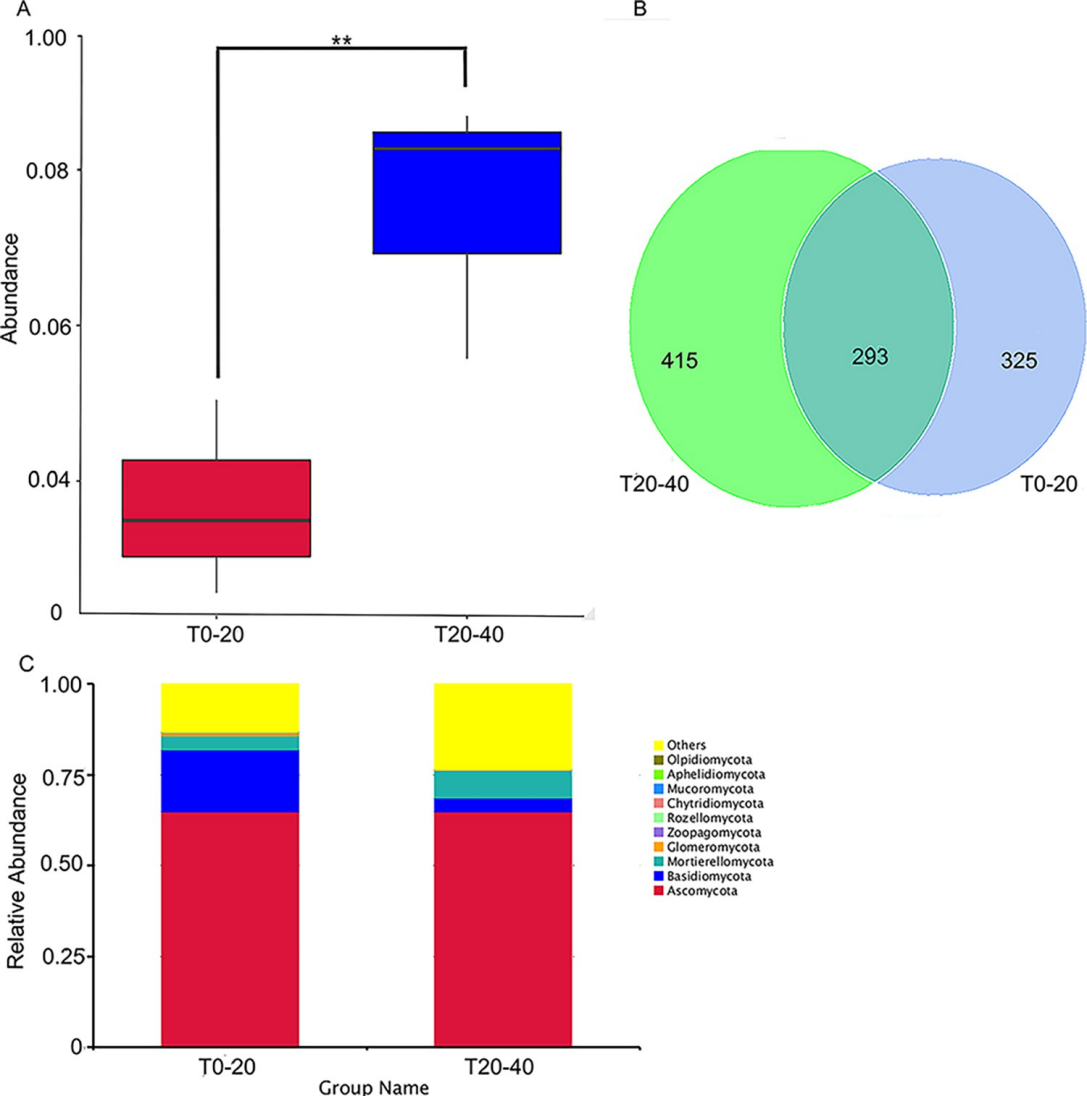
Abundance and distribution in microorganism species from T0-20 and T20-40. (A) box plot of abundance and (B) Venn graph of distribution of total microorganism species (C) relative abundance of different microorganism species among groups. The box plot was drawn according to the result of significant differences in the abundance of the different species between T0-20 and T20-40. Based on OTUs results obtained by clustering, common and unique OTUs between T0-20 and T20-40 were analyzed to form Venn graph. The numbers of circles and overlaps represent the number of OTUs shared between T0-20 and T20-40. According to the species annotation results, the top 10 species with the largest abundance in each sample at each taxonomic level (Phylum, Class, Order, Family, Genus and Species) were selected to generate the columnar accumulation chart of relative abundance of species. ‘Others’ represents the sum of the relative abundances of all other species except those 10 species. The data was obtained based on the OTUs results using soil samples, and each soil sample includes triplicates.

The abundance statistical analysis revealed a significant difference in the abundance of the microbial species between T0-20 and T20-40; the abundance in T20-40 was higher than that in T0-20 (Fig 3A). Further, we analyzed the composition changes in the microbial species in both T0-20 and T20-40 through amplicon sequencing. 1033 OTUs were identified in this study, and a total of 415 and 325 OTUs were observed in T20-40 and T0-20, respectively, excluding the 293 OTUs that did not change (Fig 3B). Ascomycota species possessed the highest relative abundance in both T0-20 and T20-40. Ascomycota, Basidiomycota, and Mortierellomycota were different between T20-40 and T0-20. Although the relative abundance of Ascomycota species in T20-40 was higher than that in T0-20, the difference was not significant compared with the difference in Basidiomycota and Mortierellomycota (Fig 3C). The data suggest that Ascomycota plays a key role in determining the soil microbial structure.

**Fig 4:**
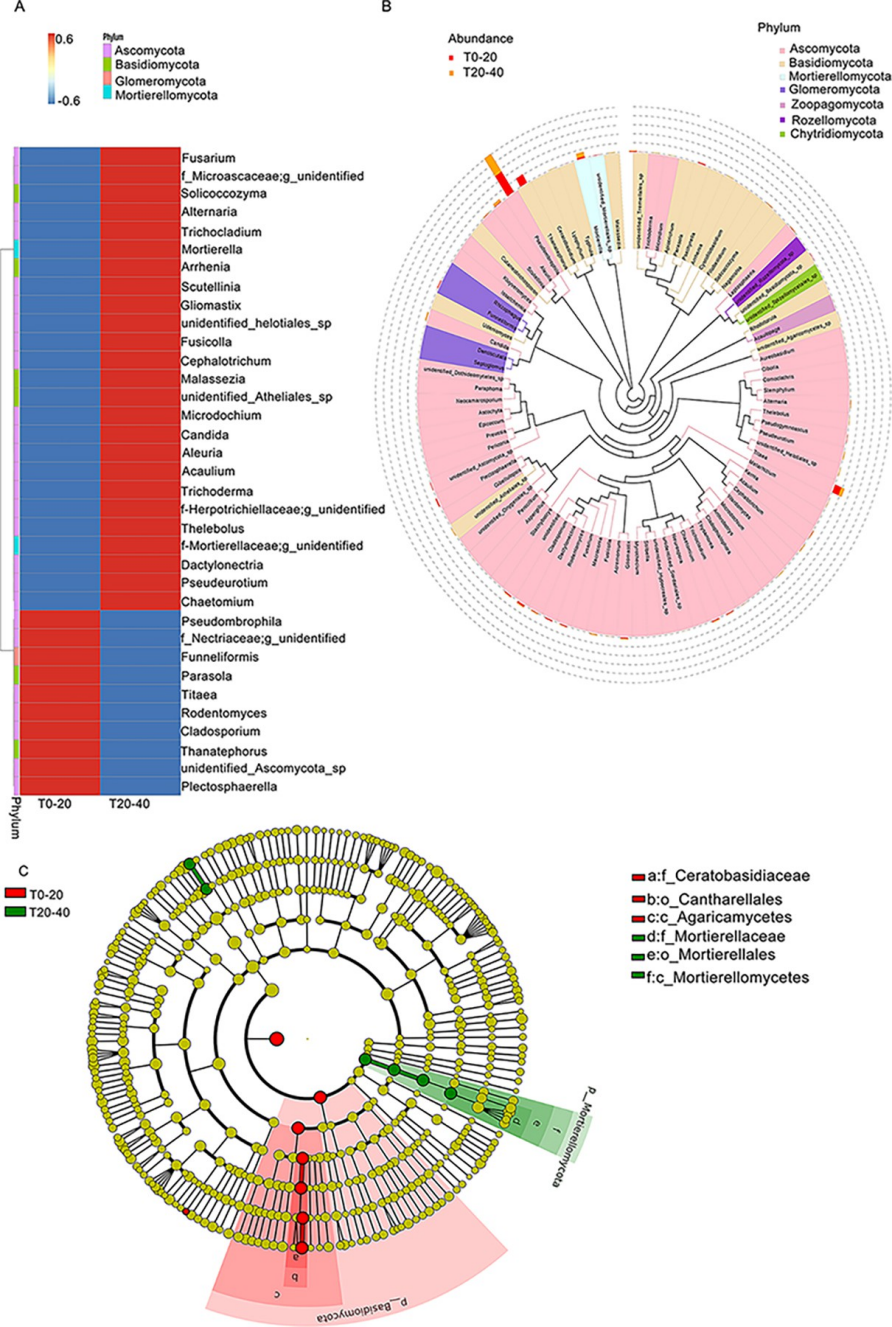
Characteristics of diversity in microorganism structure from T0-20 and T20-40. (A) heat map involved in their abundance information (B) phylogenetic tree of genus level species (C) cladogram of phylogenetic distribution based on Lefese analysis. The top 35 genera were selected at the species level according to microorganism abundance information in each sample, thereby generating heat map. Phylogenetic tree is drawn based on the representative sequences of top 100 genera obtained by multiple sequence alignment. The data was analyzed based on amplicon sequencing of soil samples, and each soil sample includes triplicates.

Within Ascomycota, the abundance of 18 species, such as Fusarium, Alternaria, Trichocladium, Scutellinia, Gliomastix, Fusicolla, Cephalotrichum, Microdochium, Candida, Aleuria, Acaulium, Trichoderma, Thelebolus, and Pseudeurotium was higher in T20-40 than that in T0-20, while only 10 species, including Titaea, Pseudombrophila, Rodentomyces, Cladosporium, and Plectosphaerella, were lesser abundant in T0-20 than that in T20-40. This indicates that the microbial species in T20-40 may be more active than that in T0-20 (Fig 4A). Among these microbial species, Pseudombrophila’s abundance was significantly higher than the other species, and Pseudombrophila from the T0-20 was highly abundant compared with that in T20-40 (Fig 4B). These observations indicate that Pseudombrophila is the primary contributor to the soil microbial community. Meanwhile, Ceratobasidiaceae (belonging to Basidiomycota) and Mortierellaceae (belonging to Mortierellomycete) were significantly different between T0-20 and T20-40 (Fig 4C).

**Fig 5:**
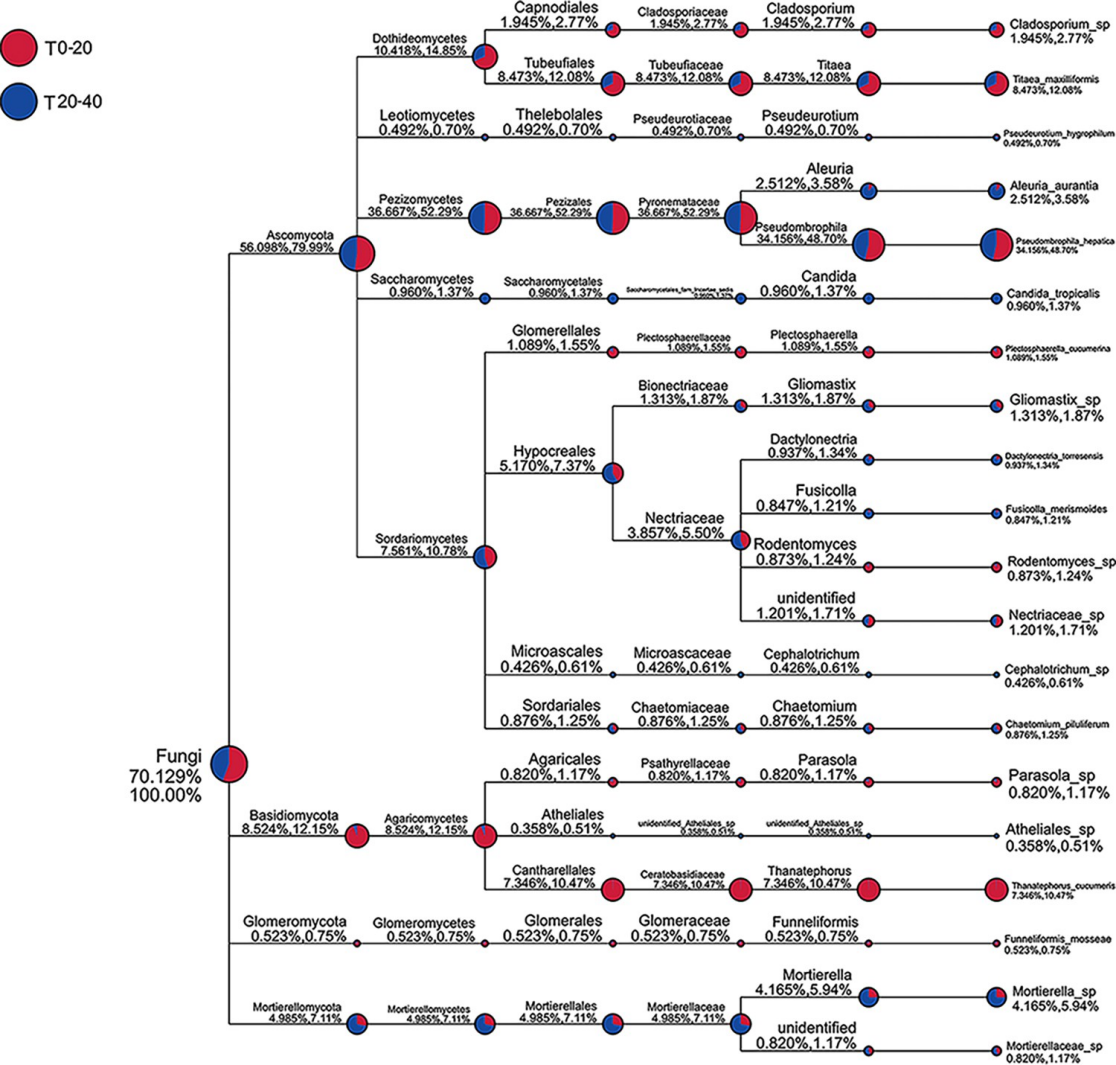
Diversity analysis of different microorganism in T0-20 and T20-40. Red and blue colors represent microorganism at 0–20 cm and 20–40 cm layers of soil. Color proportion in roundness means the percentage of different species of microorganism at different soil layers. The data was analyzed based on amplicon sequencing of soil samples, and each soil sample includes triplicates.

The proportion of 20 differential microbial species in T0-20 and T20-40 was further analyzed (Fig 5). Excluding Ceratobasidiaceae and Mortierellaceae, 18 microbial species, such as Titaea (8.43% in T0-20, 12.08% in T20-40) and Aleuria (2.51% in T0-20, 3.58% in T20-40) from Ascomyta, and Glomeraceae (0.52% in T0-20, 0.65% in T20-40) from Glomeromycota, showed the difference between T0-20 and T20-40, even though most of them were less abundant among all microbial species. Interestingly, Pseudombrophila, one of the most abundant species, showed a significant difference between T0-20 (34.2%) and T20-40 (48.7%). This observation indicates that Pseudombrophila, as the primary species, with another 19 species, determined the soil microbial diversity.

**Fig 6:**
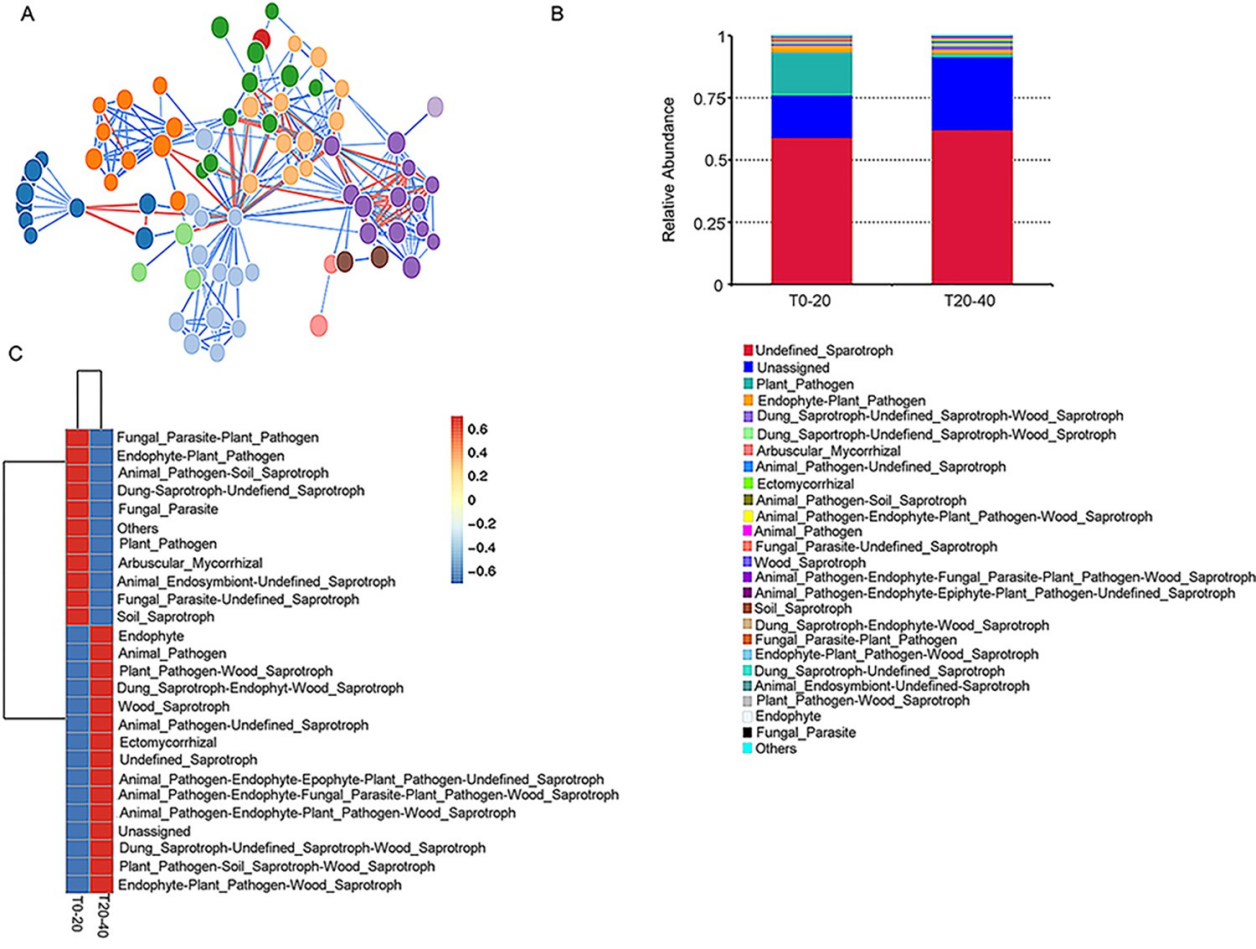
Function annotation and analysis of different microorganism species in T0-20 and T20-40. (A) Cooccurrence network diagram of the interaction in different microorganism (B) relative abundance of functional annotations and (C) heat map and clustering tree in microorganism species at different layers of soils. The cooccurrence network diagram was drawn based on the correlation coefficient of top 100 microbial genera. The top 35 functions and their abundance information in each sample were used to draw a heat map, and then clustered from the level of functional differences. The data was analyzed based on amplicon sequencing of soil samples, and each soil sample includes triplicates.

Among the microbial species observed in this study, at least 8 interacted with each other, and a close interaction was observed between the species from different phyla (Fig 6A). Although most of the microbes were not defined or assigned, a few specific plant pathogenic, soil saprotrophic, and plant endophytic species were observed in T0-20 and T20-40; the percentage in the former was higher than that in the latter (Fig 6B). In addition, 26 species showed significant differences in function between T0-20 and T20-40, and saprotrophic and plant pathogenic species showed a high proportion among these 26 species (Fig 6C).

### 3.3 Phenotype and antioxidant enzyme activities in pepper leaves infected with *P*. *capsici*

**Fig 7:**
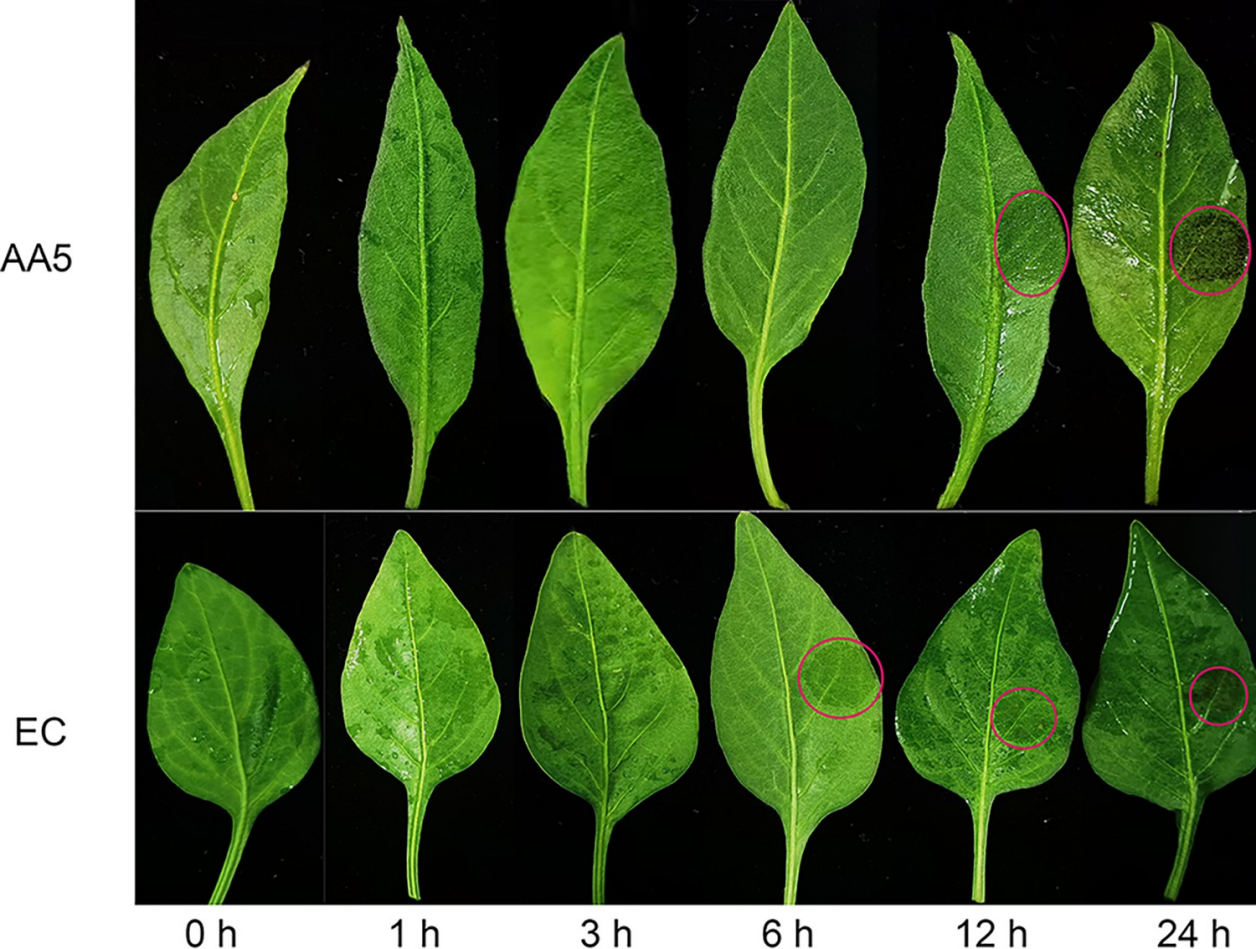
Leaf phenotype in pepper plants subjected to the infection of *P*. *capsici*. The leaves were infected using *P*. *capsici* at the 3-leafed-4-leafed stage of pepper. Samples were collected at 1 h, 3 h, 6 h, 12 h and 24 h post infection. The leaf veins were used as the dividing line, agar plugs of the media with the strain were placed on the right side of the leaf veins, while another side do same treatment with water instead of agar plugs of the media as the control. The red circle represents the site of syndrome of pepper blight. The experiment was conducted with three biological replicates, and each replicate contained three pepper seedlings.

In the next step, we isolated the virulent strain “PC-1” of *P*. *capsici* from T0-20 and T20-40, respectively, and infected the leaves of pepper cultivar “AA5” and “EC” for 2 days. Rot symptoms were observed in the leaves of pepper, which were more severe in T20-40 than in T0-20 (S2A Fig). In addition, we also found that Capsicum blight was present in the test soil due to strong saprophytic characteristics observed in the tissues/organs of pepper (S2B Fig). Fig 7 shows the pepper phenotype under Phytophthora infestation at different infection time points. No obvious change is observed on the pepper leaves within 3 h after infection compared with the control. However, 6 h after infection, the susceptible line ‘SC’ showed obvious watery spots, which became more obvious with time. Similar results were found in the resistance line ’AA5’, but the spots were found at 12 h after infection in ’AA5’ (Fig 7). These data indicate that pepper can tolerate *P*. *capsici* for a short time, and ’AA5’ is resistant compared with ’EC’.

**Fig 8:**
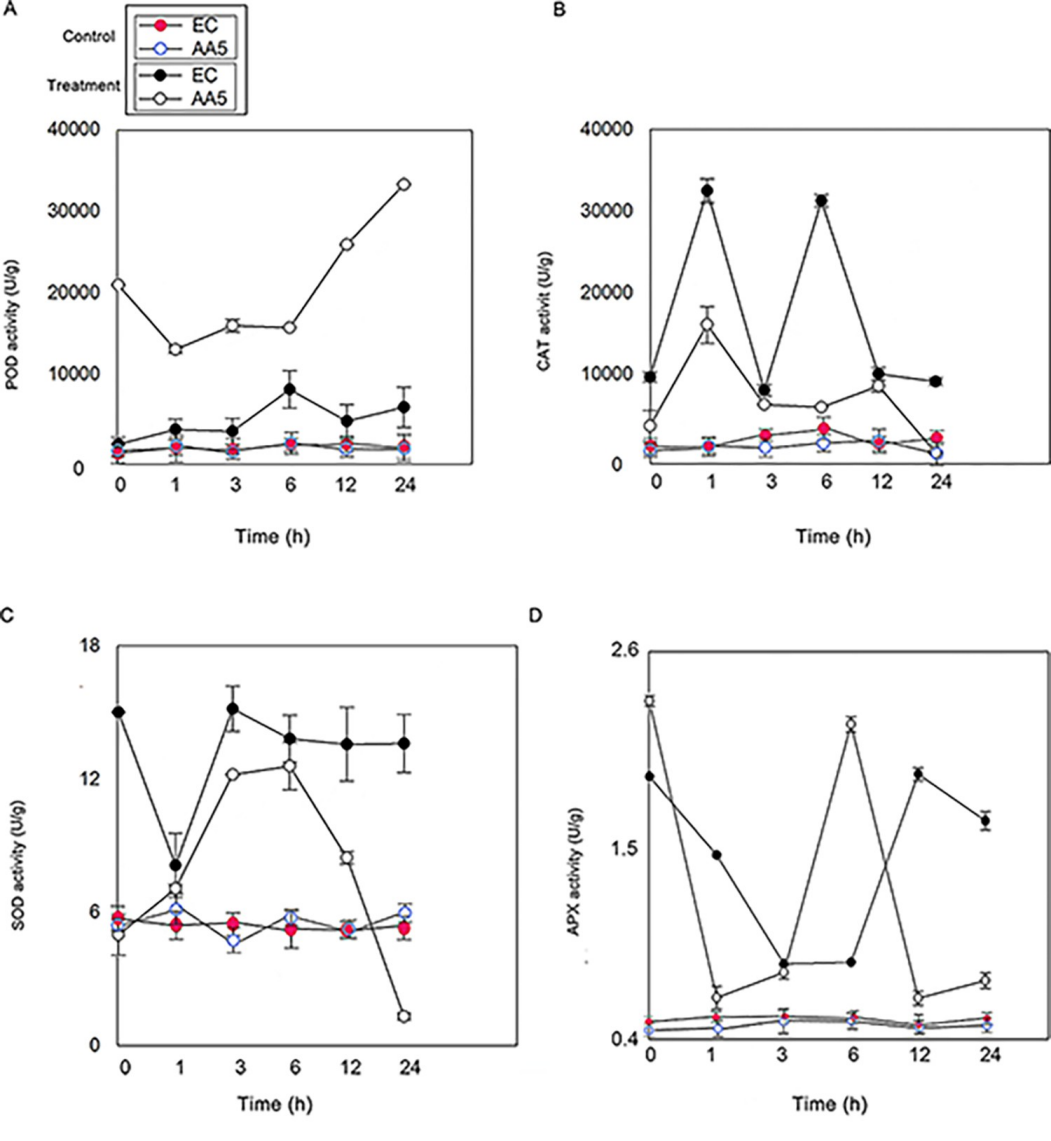
The levels of antioxidant enzymes activities. (A) POD activity (B) CAT activity (C) SOD activity (D) APX activity of pepper leaves with *P*. *capsici*. The leaves were infected using *P*. *capsici* at the 3-leafed-4-leafed stage of pepper. Samples were collected at 1 h, 3 h, 6 h, 12 h and 24 h post infection. The leaf veins were used as the dividing line, agar plugs of the media with the strain were placed on the right side of the leaf veins, while another side do same treatment with water instead of agar plugs of the media as the control. The experiment was conducted with three biological replicates, and each replicate contained three pepper seedlings.

Based on Fig 8, the activity of antioxidant enzymes was determined. Owing to treatment of no stress, the levels of all antioxidants activities showed the significant change. However, after the treatment of *P*. *capsici*, the susceptible pepper ’EC’ had higher CAT and SOD activities than the resistant pepper ’AA5’. Meanwhile, POD activity was lower in ‘EC’ than in ’AA5’ (Fig 8A). A significant decrease in the CAT and SOD activities was observed 6 h after infection in both the two treatments (Fig 8B and 8C), and no obvious change was observed in the APX activity (Fig 8D). The POD activity increased after *P*. *capsici* infection n both the two treatments, suggesting that POD plays a key role in pepper resistance.

### 3.4 Role of POD activity in prediction of *P*. *capsici*

**Fig 9:**
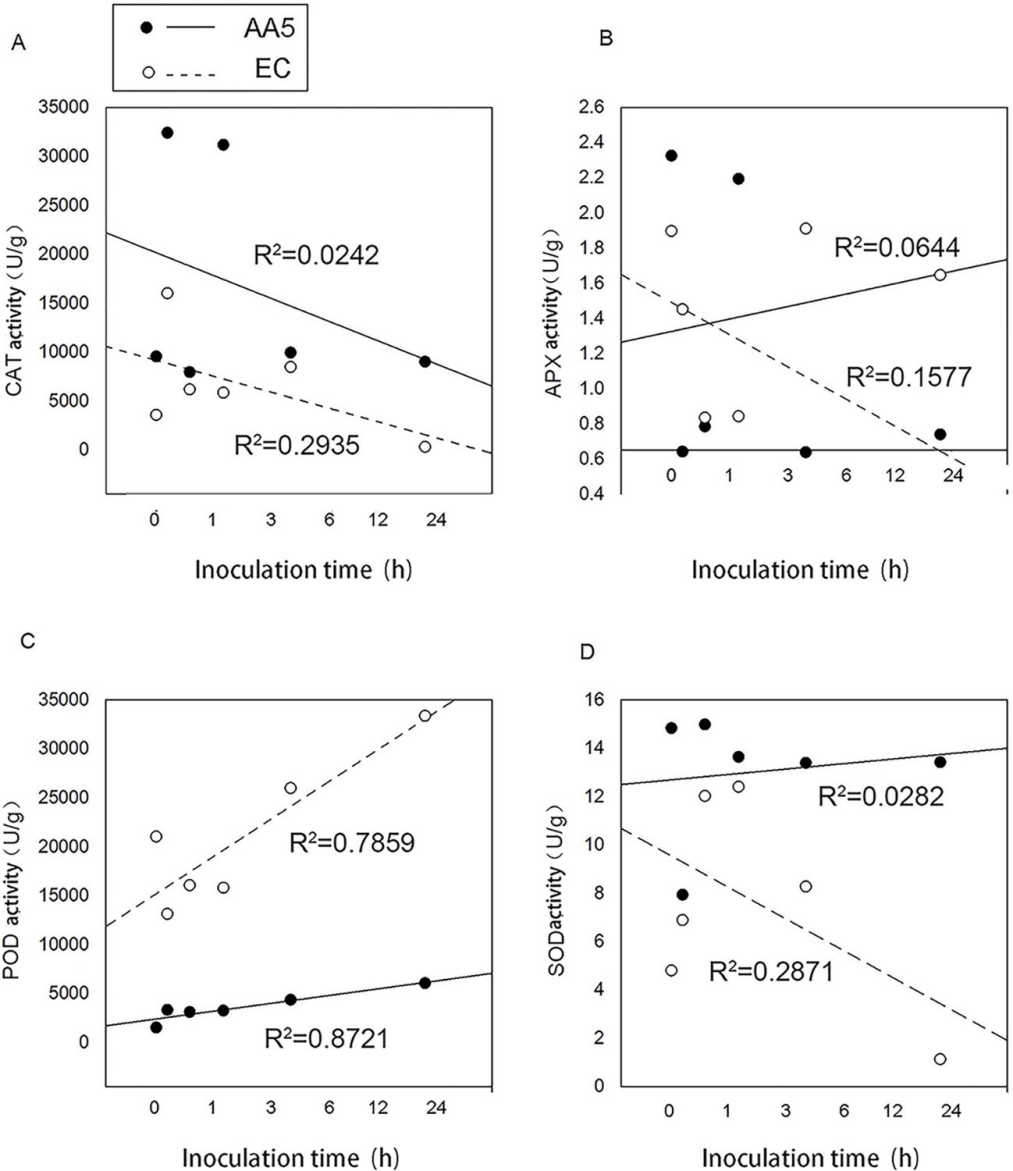
Analysis of correlation index between the activities of antioxidant enzymes and infection time R^2^ represents the correlation index, and the significant relation when R^2^ is more than 0.8. The assessment of R^2^ is based on the data from he levels of antioxidant enzyme activities.

In addition, the activities of all antioxidant enzymes, especially POD, APX, and SOD, changed sharply 3 h after infection. Importantly, after infection with *P*. *capsici*, CAT APX and SOD did show the significant relationship to the infection time due to the low correlation regression coefficient (R^2^) observed in Fig 9A, 9B and 9D. On the contrary, POD activity was positively correlated with infection time, and the R^2^ was > 0.78, and R^2^ reached 0.87 in ’EC’ (significant correlation). These observations indicate that POD was closely correlated with *P*. *capsici* infection and may be considered an indicator for predicting pepper blight (Fig 9C); moreover, 3 h was the critical time point for Phytophthora infection in pepper from an antioxidant enzyme activity perspective.

**Fig 10:**
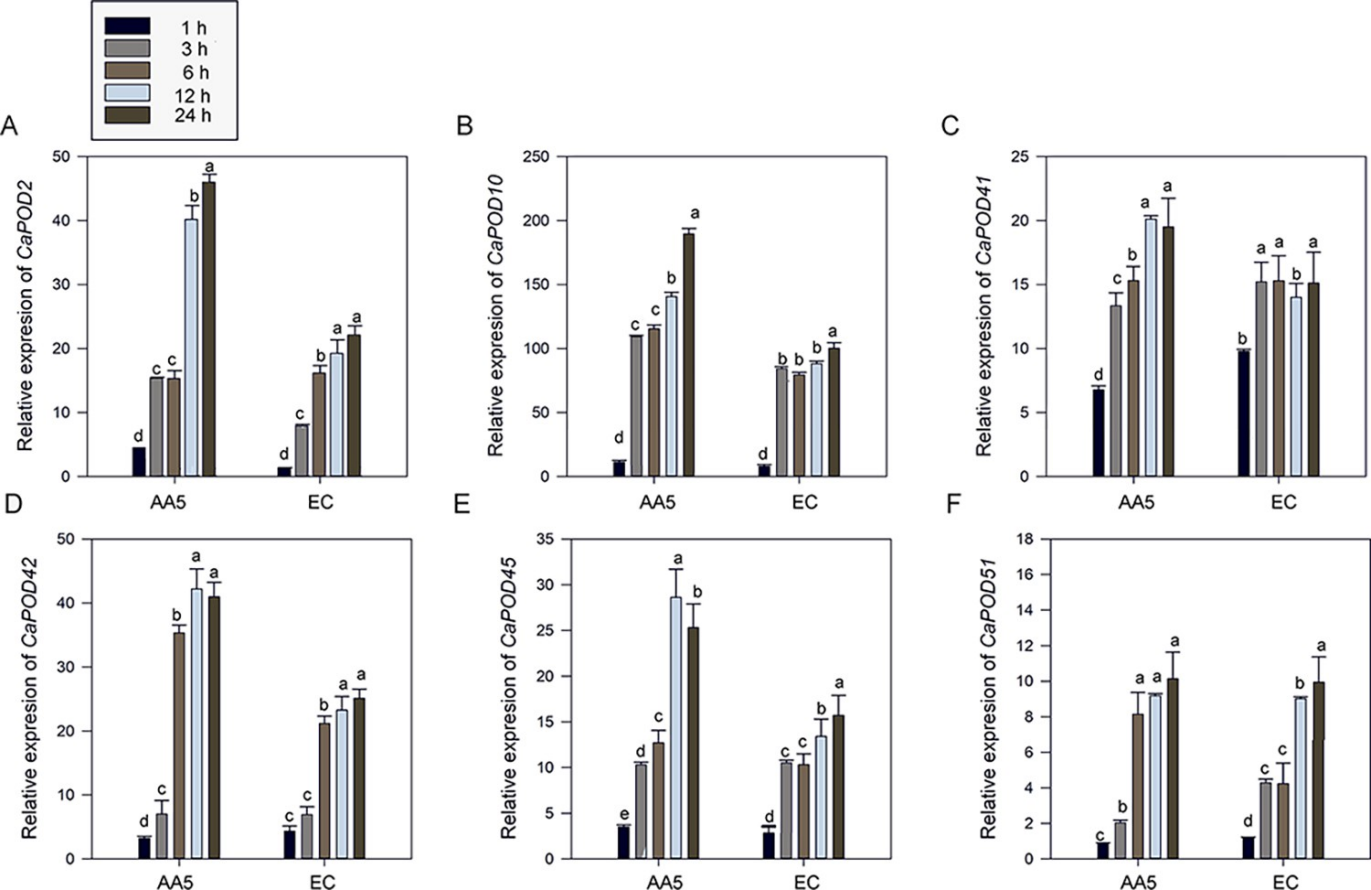
Analysis of transcriptional levels of key genes encoding POD. (A) *CaPOD2* (B) *CaPOD10* (C) *CaPOD41* (D) *CaPOD42* (E) *CaPOD45* (F) *CaPOD51* The leaves were sampled at the 4-leafed-6-leafed stage. The experiment was conducted with triplicates. The lowercase means the significance at p < 0.05 level, and the bar represented the SD of triplicates.

Further, we measured the expression levels of several genes encoding PODs. The results indicated that these genes, including *CaPOD2*, *CaPOD10*, *CaPOD41*, *CaPOD42*, *CaPOD45*, and *CaPOD51*, were significantly upregulated along with infection time progression. The pepper cultivar ‘AA5’ exhibited greater POD gene expression levels than the pepper cultivar ‘EC.’ CaPOD10 expression even reached over 150.0 at 12 h post-infection in pepper cultivar ‘AA5’ (Fig 10B). Importantly, strongly increased expression levels of these genes were observed at 3 h post-infection compared to that at 1 h post-infection, with statistically significant differences observed in gene expression between the two time points. The trend of expression of these genes was similar to that of POD activity, suggesting the accuracy of POD activity assessment in predicting *P*. *capsici* pathogenicity.

## 4 Discussion

Soil quality can be evaluated by integrating biological, chemical, and physical properties [28]. Microbial activity is a potential indicator of soil quality and can predict changes in soil properties [29]. Therefore, estimating soil microbes helps determine the soil nutrient content and components. Microorganisms decompose soil organic matter by secreting various enzymes, thereby improving the inorganic constituents of soil that are easily absorbed by plants [30, 31]. Soil enzymes produced by microorganisms transform the soil substrates via the carbon (C) and nutrient cycles [32]. Thus, soil nutrients (C and N) and pH have been significantly correlated with enzymatic activities and microbial biomass. In the current study, the changes in the soil nutrients were much more pronounced in T0-20 than in T20-40, and the changes in some soil nutrient components were statistically different, similar to the trend in the soil enzyme activities. This finding suggests that biological and chemical properties are closely related to soil depth. Importantly, a > 50% increase in UE and SC activities was observed in T0-20 compared with that in T20-40, which suggests the importance of these two microbial enzymes in forming the inorganic nutrients in the surface soil layer. UE and SC promote the formation of ammonia, glucose, and fructose in soil and provide important C and N sources for crop growth [33, 34]. This information is important for arid or saline areas of Guyuan region where soil quality is improved by adding microbial fertilizers, which enhance UE and SC activities to promote crop growth.

Researchers often used amplicon sequencing to determine microbial organisms and their interaction in soil, facilitating the identification of species and the analysis of microbial diversity [35]. Our preliminary results indicated that microbial diversity of T0-20 is better than that of T20-40, and Pseudombrophila dominates the microbial population and determines the microbial diversity together with other 19 species, such as Ceratobasidiaceae and Mortierellaceae. The existence of these microbial species plays a key role in the recycling and transforming the soil organic matter and nutrient elements and reflects the soil fertility status. Additionally, soil quality, including nutrient and enzyme activities, of surface soil, was better than that in deep soil. However, findings on Pseudombrophila are few and should be investigated in detail. In addition, during the trial, we recorded the weather data for 15 days and found that the relative humidity of only 4 days was more than 70%, and the lowest humidity was less than 20%. Correspondingly, soil water was also <20%, and no rain occurred during the growth season (S3 Table). Therefore, we speculated that less rain and excess evaporation enhanced pH and soil nutrient composition, influencing soil quality and microbial community of the surface layer much more than the deep layers.

*P*. *capsici* is an important pathogen in soil ecosystems and results in the occurrence of pepper blight [36]. In this study, leaf phenotype analysis revealed that pepper seedlings can endure *P*. *capsici* infection for a short time; the enhanced activities of antioxidant enzymes further confirmed this resistance. Studies have suggested that *P*. *capsici* substantially induces the expression of key genes, such as *CaBPM4* and *CanTF*, to improve pepper resistance to blight, which agrees with our study [37, 38]. Interestingly, we observed a substantial increase in POD activity during *P*. *capsici* infection, and a positive relationship between POD activity and the critical time of blight onset in pepper, suggesting that the POD is an indicator of *blight* occurrence. POD has antimicrobial and antioxidant properties, and changes in POD activity after pathogen infection could be used as an early indicator in screening the germplasm for blight resistance. For example, the hyperspectral signal can easily detect this change, which is important for predicting pepper blight [39]. Our study thus provides a theoretical foundation for building a pepper blight forecast model.

Water availability is a significant factor affecting mycelial growth and oospore production of pathogens [40]. In the current study, higher humidity in the deep soil layer than in the surface soil layer may be a primary reason causing severe pepper blight disease. The case is also associated with the soil carbon and nitrogen content. Improved carbon content can inhibit the development of P. capsici by enhancing the activities of beneficial microorganisms and decreasing soil nitrogen availability [41]. Amending soil with organic products enriches the carbon content and results in a proprietary mix of beneficial microorganisms and substrates, resulting in lower soil P. capsici abundance [42]. In the current study, there was no difference in available nitrogen content between T0-20 and 20–40, while a significant increase in the organic matter content is observed in T0-20 compared to T20-40. At the same time, the rot phenotype in the leaves of pepper infected by P. capsici was more severe in T20-40. Effectively managing phytophthora blight is challenging as the dispersal of sporangia via irrigation or rainwater results in the rapid movement of inoculum around crop fields, and the pathogen can rapidly kill the plants, making chemical control very difficult.

## 5 Conclusion

This study evaluated the soil quality in terms of microbial diversity, soil enzyme activity, and microbial diversity in the Loess in the Guyuan region of China and found that the 0–20 cm soil layer was suitable for crop cultivation. The topsoil layer had higher nutrient content and microbial enzyme activity due to the differences in the diversity of microorganisms between T0-20 and T20-40. Further, we identified Pseudombrophila as the primary microbial species of the soil, and this species, together with another 19 species, determined the diversity of soil microorganisms. Interestingly, the antioxidant enzyme analysis revealed that 3 h after inoculation was the critical time point for Phytophthora infection in pepper. Finally, the study proposes POD as an indicator for predicting pepper blight. These findings will provide a basis for planning strategies to prevent pepper blight in the Guyuan region.

## Supporting information

S1 Fig Collection method of soil sample. (JPG)

S2 Fig Phenotype of pepper after were infected by P. phytophthora. (JPG)

S1 Table Kits used for measurement of microorganism activity in this study. (DOCX)

S2 Table Primers used for amplicon sequencing and qRT-PCR. (DOCX)

S3 Table The weather parameters in the Guyuan region. (DOCX)

S4 Table Abbreviation list. (DOCX)

## References

[1] Huang CF, Zhou LG. A brief analysis of the living patterns of the top 20 Loess gully areas in South Ningxia based on the differential abundance ranking—A case study of Zhangyi Town, Guyuan City. Academic Conference on Chinese Dwellings; 2007:561–563.

[2] Xiao G, Zhang F, Qiu Z, Yao Y, Wang R, Huang J. Response to climate change for potato water use efficiency in semi-arid areas of China. Agri Water Manage. 2013; 127:119–23. doi: 10.1016/j.agwat.2013.06.004

[3] Xu S-J, Pan B-T, Gao H-S, Cao G-J, Su H. Changes in sand fractions of Binggou section and the expansion and contraction of the tengger desert during 50–30 ka. Earth Surf Proc Lands. 2007; 32(3):475–480. 10.1002/esp.1439

[4] Chen S, Jin Z, Zhang J, Yang S. Soil quality assessment in different dammed-valley farmlands in the hilly-gully mountain areas of the northern Loess Plateau, China. J Arid Land. 2021; 13(8):777–789. 10.1007/s40333-021-0014-4

[5] Zhang F, Zhao C, Lourenco SDN, Dong S, Jiang Y. Factors affecting the soil-water retention curve of Chinese loess. B Eng Geol Environ. 2021; 80(1):717–729. 10.1007/s10064-020-01959-9

[6] Ochoa-Hueso R, Delgado-Baquerizo M, Gallardo A, Bowker MA, Maestre FT. Climatic conditions, soil fertility and atmospheric nitrogen deposition largely determine the structure and functioning of microbial communities in biocrust-dominated Mediterranean drylands. Plant Soil. 2016; 399(1–2):271–282. 10.1007/s11104-015-2695-y

[7] Young KE, Ferrenberg S, Reibold R, Reed SC, Swenson T, Northen T, et al. Vertical movement of soluble carbon and nutrients from biocrusts to subsurface mineral soils. Geoderma. 2022; 405:115495. 10.1016/j.geoderma.2021.115495

[8] Zhan X, Wu W, Zhou L, Liang J, Jiang T. Interactive effect of dissolved organic matter and phenanthrene on soil enzymatic activities. J Environ Sci. 2010; 22(4):607–614. doi: 10.1016/s1001-0742(09)60139-x 20617739

[9] Zhang Y, Chen L, Wu Z, Sun C. Kinetic parameters of soil beta-glucosidase response to environmental temperature and moisture regimes. Rev Bras Cien Solo. 2011; 35(4):1285–1291. 10.1590/s0100-06832011000400022

[10] Xie W, Yang J, Gao S, Yao R, Wang X. The effect and influence mechanism of soil salinity on phosphorus availability in coastal salt-affected soils. Water. 2022; 14(18):2804. 10.3390/w14182804

[11] Hao Z, Ye CL, Yi W, Hui G, Hu SJJPS. Characteristics of soil microbial respiration and its response to temperature change in different soil depths in Yunwu Mountain grassland. Pratacultural Sci. 2017; 34(2):224–230. 10.11829/j.issn.1001-0629.2016-0278

[12] Yang XZ, Chen LJ. Distribution of exogenous phytase activity in soil solid-liquid phases and their effect on soil organic P hydrolysis. J Plant Nutr Soil Sci. 2017; 180(1):39–48. ttps://doi.org/10.1002/jpln.201600421

[13] Zheng MM, Wang C, Li WX, Guo L, Cai ZJ, Wang BR, et al. Changes of acid and alkaline phosphatase activities in long-term chemical fertilization are driven by the similar soil properties and associated microbial community composition in acidic soil. Eur J Soil Bioly. 2021; 104:103312. 10.1016/j.ejsobi.2021.103312

[14] Lee YM, Chae GY, Kim MK, Kim S. Comparative analysis of re-annotated genes provides insight into evolutionary divergence and expressions of aquaporin family in pepper. Plants-Basel. 2021; 10(6):1039. doi: 10.3390/plants10061039 34064088 PMC8224332

[15] Qin C, Yu C, Shen Y, Fang X, Chen L, Min J, et al. Whole-genome sequencing of cultivated and wild peppers provides insights into Capsicum domestication and specialization. P Natl Acad Sci.USA. 2014; 111(4):5135–5140. doi: 10.1073/pnas.1400975111 24591624 PMC3986200

[16] Babadoost M, Pavon C, Islam SZ, Tian D, editors. Phytophthora blight (*Phytophthora capsic*i) of pepper and its management. Acta Horticulturae Sinica. 2015; 1105:61–66. 10.17660/ActaHortic.2015.1105.9

[17] Li M, Cai P, Hou S, Cheng Z, Wu F, Lin X, et al. Degradation of soil arbuscular mycorrhizal fungal diversity and functionality accompanied by the aggravation of pepper Phytophthora blight in a facility shed in Southwest China. Land Degrad Dev. 2022; 33(9):1337–1346. 10.1002/ldr.4228

[18] Wan JSH, Liew ECY. Efficacy of chemical and biological agents against pepper blight (*Phytophthora capsici* Leonion) in East Asia: a meta-analysis of laboratory and field trial data. J Plant Pathol. 2020; 102(3):835–842. 10.1007/s42161-020-00519-0

[19] Yeon S-J, Kim J-H, Cho W-Y, Kim S-K, Seo HG, Lee C-H. In vitro studies of fermented korean chung-yang hot pepper phenolics as inhibitors of key enzymes relevant to hypertension and diabetes. Foods. 2019; 8(10):498. doi: 10.3390/foods8100498 31615144 PMC6835475

[20] Warris A, Ballou ER. Oxidative responses and fungal infection biology. Sem Cell Dev Biol. 2019; 89:34–46. doi: 10.1016/j.semcdb.2018.03.004 29522807

[21] Ramzan M, Sana S, Javaid N, Shah AA, Ejaz S, Malik WN, et al. Mitigation of bacterial spot disease induced biotic stress in *Capsicum annuum* L. cultivars via antioxidant enzymes and isoforms. Sci Rep. 2021; 11(1):9445. doi: 10.1038/s41598-021-88797-1 33941790 PMC8093210

[22] Mak WC, Chan CY, Barford J, Renneberg R. Biosensor for rapid phosphate monitoring in a sequencing batch reactor (SBR) system. Biosens Bioelectron. 2003; 19(3):233–237. doi: 10.1016/s0956-5663(03)00209-4 14611759

[23] Wang J, Tian T, Wang H, Cui J, Zhu Y, Zhang W, et al. Estimating cotton leaf nitrogen by combining the bands sensitive to nitrogen concentration and oxidase activities using hyperspectral imaging. Comput Electron Agr. 2021; 189:106390. 10.1016/j.compag.2021.106390

[24] Devdas D, Srivastava LK, Verma U. Study on correlation between physico-chemical properties of ph, oc and available n, p and k in black soil of navagarh block under janjgir district in chhattisgarh. An Asian Journal of Soil Science. 2013; 8(2):381–384.

[25] Stefanova P, Taseva M, Georgieva T, Gotcheva V, Angelov A. A modified ctab method for dna extraction from soybean and meat products. Biotechnol Biotec E. 2013; 27(3):3803–3810. 10.5504/bbeq.2013.0026

[26] Zhang HX, Jin JH, He YM, Lu BY, Li DW, Chai WG, et al. Genome-wide identification and analysis of the SBP-Box family genes under *Phytophthora capsici* stress in pepper (*Capsicum annuum* L.). Front Plant Sci. 2016; 7:504. doi: 10.3389/fpls.2016.00504 27148327 PMC4832253

[27] Dionisio-Sese ML, Tobita S. Antioxidant responses of rice seedlings to salinity stress. Plant Sci. 1998; 135(1):1–9. 10.1016/S0168-9452(98)00025-9

[28] Pell M, Stenstrom JJB. Evaluation and characterization of soil microbiological processes. In book: Soil-water-solute process characterization. 2005; 559–584. 10.1201/9781420032086.sec4

[29] Rose CM. The effect of inorganic and organic soil amendments on soil microbial activity assessed via multiple microbial assays: Salisbury University.; 2014.

[30] Xu Z, Yu G, Zhang X, Ge J, He N, Wang Q, et al. The variations in soil microbial communities, enzyme activities and their relationships with soil organic matter decomposition along the northern slope of Changbai Mountain. Appl Soil Ecol. 2015; 86:19–29. 10.1016/j.apsoil.2014.09.015

[31] Schuetz K, Kandeler E, Nagel P, Scheu S, Ruess L. Functional microbial community response to nutrient pulses by artificial groundwater recharge practice in surface soils and subsoils. Fems Microbiology Ecology. 2010; 72(3):445–455. doi: 10.1111/j.1574-6941.2010.00855.x 20557572

[32] Jing X, Chen X, Fang J, Ji C, Shen H, Zheng C, et al. Soil microbial carbon and nutrient constraints are driven more by climate and soil physicochemical properties than by nutrient addition in forest ecosystems. Soil Biol Biochem. 2020; 141:107657. 10.1016/j.soilbio.2019.107657

[33] Salehuddin NF, Mansor N, Yahya WZN, Affendi NMN, Manogaran MD. Organosulfur compounds as soil urease inhibitors and their effect on kinetics of urea hydrolysis. J Soil Sci Plant Nut. 2021; 21(4):2652–2659. 10.1007/s42729-021-00553-6

[34] Zhang Y, Cui D, Yang H, Kasim N. Differences of soil enzyme activities and its influencing factors under different flooding conditions in Ili Valley, Xinjiang. Peerj. 2020; 8:e8531. doi: 10.7717/peerj.8531 32201637 PMC7073240

[35] Golebiewski M, Tretyn A. Generating amplicon reads for microbial community assessment with next-generation sequencing. J App Microbiol. 2020; 128(2):330–354. doi: 10.1111/jam.14380 31299126

[36] Ro N, Haile M, Hur O, Geum B, Rhee J, Hwang A, et al. Genome-wide association study of resistance to phytophthora capsici in the pepper (Capsicum spp.) collection. Fronti Plant Sci. 2022; 13:902464. doi: 10.3389/fpls.2022.902464 35668797 PMC9164128

[37] He Y-M, Liu K-K, Zhang H-X, Cheng G-X, Ali M, Ul Haq S, et al. Contribution of CaBPM4, a BTB domain-containing gene, to the response of pepper to *Phytophthora capsici* infection and abiotic stresses. Agronomy-Basel. 2019; 9(8):417. 10.3390/agronomy9080417

[38] He Y-M, Luo D-X, Khan A, Liu K-K, Arisha MH, Zhang H-X, et al. CanTF, a novel transcription factor in pepper, is involved in resistance to *Phytophthora capsici* as well as abiotic stresses. Plant Mol Biol Rep. 2018; 36(5–6):776–789. 10.1007/s11105-018-1121-z

[39] Wu L, Jiang Q, Zhang Y, Du M, Ma L, Ma Y. Peroxidase activity in tomato leaf cells under salt stress based on micro-hyperspectral imaging technique. Horticulturae. 2022; 8(9):813. 10.3390/horticulturae8090813

[40] Sanogo S, Ji P. Water management in relation to control of *Phytophthora capsici* in vegetable crops. Agr Water Manage. 2013; 129:113–119. 10.1016/j.agwat.2013.07.018

[41] Kim YS, Jang BR, Chung IM, Sang MK, & Chun SC. Enhancement of biocontrol activity of antagonistic chryseobacterium strain kj1r5 by adding carbon sources against *Phytophthora capsici*. Plant Pathol J. 2008; 24(2):164–170. 10.5423/PPJ.2008.24.2.164

[42] Shoaf N, Hoagland L, Egel DS. Suppression of phytophthora blight in sweet pepper depends on biochar amendment and soil type. HortScience: a publication of the American Society for Horticultural Science. 2016; 51(5):518–524. 10.21273/HORTSCI.51.5.518

