# Supplementary figures and images for: Assessment of soil property in the Guyuan region from Ningxia Province of China and prediction of pepper blight
Source: PLoS One. 2023 Nov 20;18(11):e0293173. doi: 10.1371/journal.pone.0293173 (PMC10659199; doi:10.1371/journal.pone.0293173)

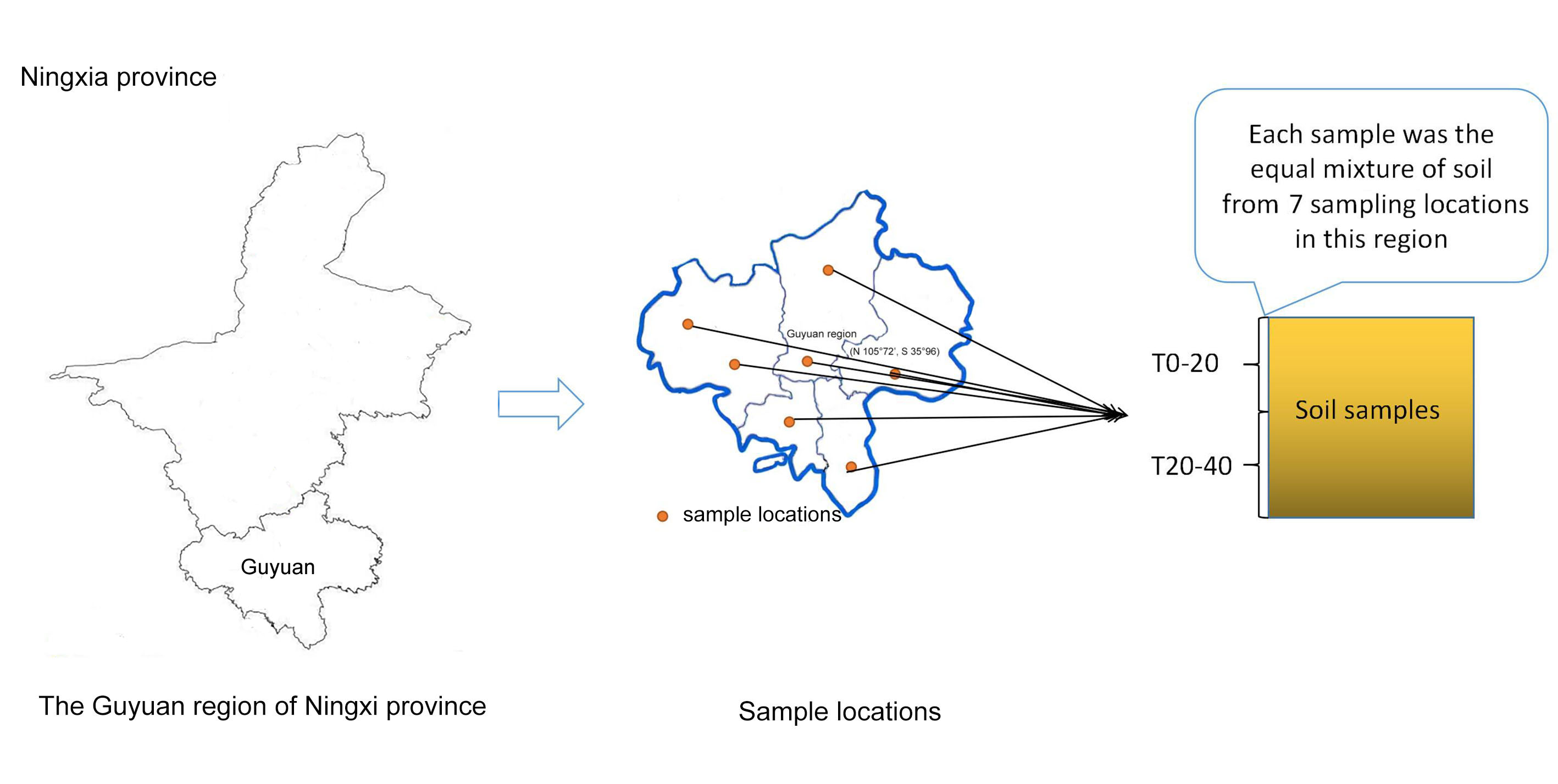

Supplement: S1 Fig — (JPG) [file pone.0293173.s001.jpg]

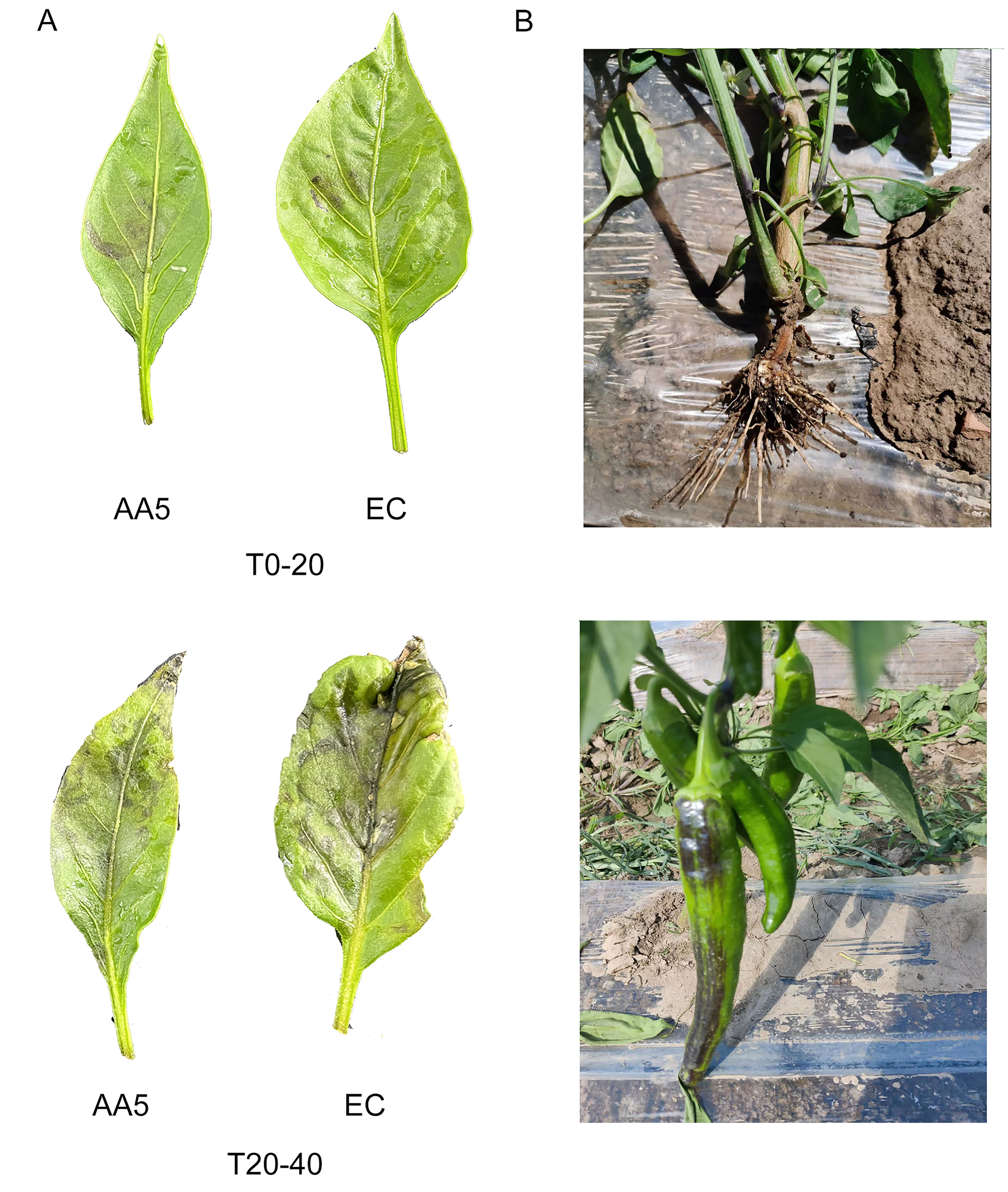

Supplement: S2 Fig — (JPG) [file pone.0293173.s002.jpg]
