# Supplementary material for: Assessment of soil property in the Guyuan region from Ningxia Province of China and prediction of pepper blight
Source: PLoS One. 2023 Nov 20;18(11):e0293173. doi: 10.1371/journal.pone.0293173 (PMC10659199; doi:10.1371/journal.pone.0293173)
Supplement: S1 Table — (DOCX) [file pone.0293173.s003.docx]

| **S1 Table kits used for measurement of microorganism activity in this study** | | | |
| --- | --- | --- | --- |
| **Contributed parameters** | **Kit names** | **Produstion Type** | **Manufacturer** |
| UE | Solid-Urease kit | JC0901-M | JICE Biotechnology Co., LTD, Nanjing, China |
| PPO | Solid-Polyphenol oxidase kit | JC0903-S |  |
| α-GC | Solid-α-Glucosidase kit | JC0913-M |  |
| β-GC | Solid-β-Glucosidase kit | JC0904-M |  |
| CL | Solid-cellulase kit | JC0905-M |  |
| CAT | Solid-Catalase kit | JC0902-M |  |
| SC | Solid-Sucrase kit | JC0907-M |  |
| POD | Solid- Peroxidase kit | JC0911-S） |  |
| AKP/ALP | Soil alkaline phosphatase kit | JC0917-M |  |
| ALPT | Solid - Alcalase Protease kit | JC0910-M |  |
