# Supplementary material for: Assessment of soil property in the Guyuan region from Ningxia Province of China and prediction of pepper blight
Source: PLoS One. 2023 Nov 20;18(11):e0293173. doi: 10.1371/journal.pone.0293173 (PMC10659199; doi:10.1371/journal.pone.0293173)
Supplement: S2 Table — (DOCX) [file pone.0293173.s004.docx]

| **S2 Table primers used for amplicon sequencing in the microorganism of soil** | |
| --- | --- |
| **Primer name** | **Sequence (5’——3’)** |
| 505F | GTGCCAGCMGCCGCGG |
| 806R | GGACTACHVGGGTWTCTAAT |
| 528F | GCCTCCCTCGCGCCATCAGGCGGTAATTCCAGCTCCAA |
| 706R | GCCTTGCCAGCCCGATCAGAATCCRAGAATTTCACCTCT |
| ITS1 | TCCGTAGGTGAACCTGCGG |
| ITS4 | TCCTCCGCTTATTGGATATGC |
| CaPOD2F | AGAAAGAAAGTCAGCCAAAT |
| CaPOD2R | AATACCTCCATCACAACCAT |
| CaPOD10F | ATTACACTTGTCCCAACCTC |
| CaPOD10R | ATTAGGAATTGCGTTCTTCT |
| CaPOD41F | ACGACATGAACCATTCCCTC |
| CaPOD41R | GCTGTCTTTCCTCCCTAACG |
| CaPOD42F | TCCAAAGGCTGTGCAGTATGT |
| CaPOD42R | AGGGCTTAGTCCTCTTGTCAGT |
| CaPOD45F | TCCTCCTCCTACTTTCTATCA |
| CaPOD45R | AGCATCACAACCCTGAATAA |
| CaPOD51F | TCCCTGCTGTTCTTCGTCTC |
| CaPOD51R | CAAATCCATCTCCTGCCAAT |
