# Supplementary material for: Assessment of soil property in the Guyuan region from Ningxia Province of China and prediction of pepper blight
Source: PLoS One. 2023 Nov 20;18(11):e0293173. doi: 10.1371/journal.pone.0293173 (PMC10659199; doi:10.1371/journal.pone.0293173)
Supplement: S3 Table — (DOCX) [file pone.0293173.s005.docx]

| **S3 Table. The weather parameters in the Guyuan region** | | | | | | | | | | |
| --- | --- | --- | --- | --- | --- | --- | --- | --- | --- | --- |
| Days | **Temperature** | | | **Relative humidity (%)** | | | **The maximum light intensity (lx)** | **Soil water (%)** | | |
|  | **Mix** | **Min** | **Max-Min** | **Max** | **Min** | **Mean** |  | **Max** | **Min** | **Mean** |
| 1 | 28.29 | 0.00 | 28.29 | 56.26 | 0.00 | 37.70 | 1,052 | 22.42 | 0.00 | 5.92 |
| 2 | 36.65 | 12.66 | 23.99 | 73.87 | 17.99 | 56.02 | 29,632 | 13.51 | 0.45 | 5.45 |
| 3 | 33.72 | 14.28 | 19.44 | 71.70 | 26.03 | 55.85 | 29,348 | 9.46 | 4.07 | 7.28 |
| 4 | 35.54 | 17.80 | 17.74 | 70.56 | 22.42 | 53.40 | 27,601 | 10.06 | 3.87 | 6.18 |
| 5 | 36.29 | 18.20 | 18.09 | 66.04 | 29.45 | 53.01 | 25,683 | 16.78 | 5.33 | 8.72 |
| 6 | 35.23 | 18.20 | 17.03 | 70.21 | 30.13 | 57.38 | 26,868 | 19.42 | 6.41 | 12.76 |
| 7 | 31.58 | 18.62 | 12.96 | 74.73 | 31.99 | 56.88 | 23,742 | 18.09 | 13.06 | 14.67 |
| 8 | 32.28 | 19.98 | 12.3 | 89.75 | 42.13 | 70.07 | 9,595 | 20.08 | 12.18 | 16.12 |
| 9 | 32.38 | 19.87 | 12.51 | 92.54 | 53.38 | 79.47 | 31,165 | 21.75 | 13.04 | 15.10 |
| 10 | 34.87 | 18.86 | 16.01 | 92.39 | 48.74 | 75.89 | 49,107 | 14.59 | 7.40 | 11.22 |
| 11 | 36.54 | 16.73 | 19.81 | 89.52 | 41.39 | 71.12 | 37,360 | 7.42 | 0.00 | 3.92 |
| 12 | 39.08 | 18.14 | 20.94 | 80.67 | 30.77 | 59.75 | 33,781 | 3.43 | 0.00 | 0.70 |
| 13 | 32.57 | 18.13 | 14.44 | 84.31 | 41.76 | 65.75 | 30,012 | 2.05 | 0.26 | 0.68 |
| 14 | 28.50 | 19.08 | 9.42 | 84.61 | 51.29 | 68.95 | 18,082 | 23.19 | 0.87 | 12.87 |
| 15 | 30.22 | 17.62 | 12.6 | 87.67 | 36.36 | 63.72 | 30,051 | 22.79 | 16.61 | 18.19 |
| 16 | 33.58 | 15.57 | 18.01 | 84.70 | 38.71 | 63.32 | 36,744 | 23.83 | 16.49 | 18.41 |
| 17 | 33.03 | 15.64 | 17.39 | 84.87 | 41.63 | 70.63 | 28,155 | 22.81 | 14.83 | 17.83 |
| 18 | 31.82 | 16.72 | 15.1 | 85.58 | 36.56 | 61.58 | 27,710 | 22.08 | 14.29 | 16.37 |
| 19 | 31.83 | 18.36 | 13.47 | 80.81 | 38.82 | 64.41 | 22,761 | 21.73 | 14.99 | 16.71 |
