# Supplementary material for: Assessment of soil property in the Guyuan region from Ningxia Province of China and prediction of pepper blight
Source: PLoS One. 2023 Nov 20;18(11):e0293173. doi: 10.1371/journal.pone.0293173 (PMC10659199; doi:10.1371/journal.pone.0293173)
Supplement: S4 Table — (DOCX) [file pone.0293173.s006.docx]

| **S4 Table** **Abbreviations list** | |
| --- | --- |
| **Abbreviations** | **Full name** |
| T0-20 | The 0–20 cm soil layer |
| T20-40 | The 20–40 cm soil layer |
| β-GC | β-grape-glycosidase |
| SC | sucrase |
| UE | urease |
| PPO | polyphenol oxidase |
| α-GC | α-glucosidase |
| CL | cellulase |
| CAT | catalase |
| POD | peroxidase |
| AKP/ALP | alkaline phosphatase |
| ALPT | alkaline phosphatase |
| *Phytophthora capsici* | *P. capsici* |
| ROS | oxygen species |
| SOD | superoxide dismutase |
| CAT | catalase |
| APX | ascorbate oxidase |
| CTAB | cetyl trimethyl ammonium bromide |
| MDA | malondialdehyde |
| SD | standard deviation |
| N | nitrogen |
| P | phosphorus |
| K | kalium |
| OTUs | Operational Taxonomic Units |
| PDA | potato dextrose agar |
| ODs | optical densities |
